# Reports of Guillain-Barré Syndrome After COVID-19 Vaccination in the United States

**DOI:** 10.1001/jamanetworkopen.2022.53845

**Published:** 2023-02-01

**Authors:** Winston E. Abara, Julianne Gee, Paige Marquez, Jared Woo, Tanya R. Myers, Allison DeSantis, Jane A. G. Baumblatt, Emily Jane Woo, Deborah Thompson, Narayan Nair, John R. Su, Tom T. Shimabukuro, David K. Shay

**Affiliations:** 1 COVID-19 Response Team, Centers for Disease Control and Prevention, Atlanta, Georgia; 2 Office of Biostatistics and Epidemiology, Center for Biologics Evaluation and Research, Food and Drug Administration, Silver Spring, Maryland

## Abstract

**Importance:**

Because of historical associations between vaccines and Guillain-Barré syndrome (GBS), the condition was a prespecified adverse event of special interest for COVID-19 vaccine monitoring.

**Objective:**

To evaluate GBS reports to the Vaccine Adverse Event Reporting System (VAERS) and compare reporting patterns within 21 and 42 days after vaccination with Ad26.COV2.S (Janssen), BNT162b2 (Pfizer-BioNTech), and mRNA-1273 (Moderna) COVID-19 vaccines.

**Design, Setting, and Participants:**

This retrospective cohort study was conducted using US VAERS reports submitted during December 2020 to January 2022. GBS case reports verified as meeting the Brighton Collaboration case definition for GBS in US adults after COVID-19 vaccination were included.

**Exposures:**

Receipt of the Ad26.COV2.S, BNT162b2, or mRNA-1273 COVID-19 vaccine.

**Main Outcomes and Measures:**

Descriptive analyses of GBS case were conducted. GBS reporting rates within 21 and 42 days after Ad26.COV2.S, BNT162b2, or mRNA-1273 vaccination based on doses administered were calculated. Reporting rate ratios (RRRs) after receipt of Ad26.COV2.S vs BNT162b2 or mRNA-1273 within 21- and 42-day postvaccination intervals were calculated. Observed-to-expected (OE) ratios were estimated using published GBS background rates.

**Results:**

Among 487 651 785 COVID-19 vaccine doses, 17 944 515 doses (3.7%) were Ad26.COV2.S, 266 859 784 doses (54.7%) were BNT162b2, and 202 847 486 doses (41.6%) were mRNA-1273. Of 295 verified reports of individuals with GBS identified after COVID-19 vaccination (12 Asian [4.1%], 18 Black [6.1%], and 193 White [65.4%]; 17 Hispanic [5.8%]; 169 males [57.3%]; median [IQR] age, 59.0 [46.0-68.0] years), 275 reports (93.2%) documented hospitalization. There were 209 and 253 reports of GBS that occurred within 21 days and 42 days of vaccination, respectively. Within 21 days of vaccination, GBS reporting rates per 1 000 000 doses were 3.29 for Ad26.COV.2, 0.29 for BNT162b2, and 0.35 for mRNA-1273 administered; within 42 days of vaccination, they were 4.07 for Ad26.COV.2, 0.34 for BNT162b2, and 0.44 for mRNA-1273. GBS was more frequently reported within 21 days after Ad26.COV2.S than after BNT162b2 (RRR = 11.40; 95% CI, 8.11-15.99) or mRNA-1273 (RRR = 9.26; 95% CI, 6.57-13.07) vaccination; similar findings were observed within 42 days after vaccination (BNT162b2: RRR = 12.06; 95% CI, 8.86-16.43; mRNA-1273: RRR = 9.27; 95% CI, 6.80-12.63). OE ratios were 3.79 (95% CI, 2.88-4.88) for 21-day and 2.34 (95% CI, 1.83-2.94) for 42-day intervals after Ad26.COV2.S vaccination and less than 1 (not significant) after BNT162b2 and mRNA-1273 vaccination within both postvaccination periods.

**Conclusions and Relevance:**

This study found disproportionate reporting and imbalances after Ad26.COV2.S vaccination, suggesting that Ad26.COV2.S vaccination was associated with increased risk for GBS. No associations between mRNA COVID-19 vaccines and risk of GBS were observed.

## Key Points

**Question:**

Are Ad26.COV2.S (Janssen), BNT162b2 (Pfizer-BioNTech), or mRNA-1273 (Moderna) COVID-19 vaccines associated with Guillain-Barré syndrome (GBS) within 21 or 42 days after vaccination?

**Findings:**

This cohort study of 487 651 785 COVID-19 vaccine doses found that in observed-to-expected analyses, the observed number of GBS reports was higher than expected based on background rates within 21 and 42 days after vaccination for Ad26.COV2.S but not BNT162b2 or mRNA-1273. GBS reporting rates within 21 and 42 days of Ad26.COV2.S vaccination were 9 to 12 times higher than after BNT162b2 or mRNA-1273 vaccination.

**Meaning:**

These findings suggest that Ad26.COV2.S vaccination was associated with GBS and that GBS after BNT162b2 and mRNA-1273 may represent background incidence.

## Teaser

This cohort study investigates the association of Ad26.COV2.S, BNT162b2, and mRNA-1273 COVID-19 vaccination with rates of Guillain-Barré syndrome among US adults.

## Introduction

Guillain-Barré syndrome (GBS) is a rare, immune-mediated neurologic disorder of the peripheral nervous system that is characterized by ascending weakness and paralysis.^1^ Between 3000 and 6000 cases of GBS (1-2 cases/100 000 persons) are diagnosed annually in the United States.^2,3^ Although the etiology of GBS is unclear, molecular mimicry is thought to play a role in its pathogenesis.^4^ Autoimmune antibodies likely target epitopes on peripheral nerves, leading to neuronal demyelination and axonal damage.^4^ Production of these autoimmune antibodies may be triggered by an antecedent infection. Approximately two-thirds of patients with GBS report an antecedent gastrointestinal or respiratory infection within 3 weeks before symptom onset.^5^ Rarely, vaccination has been associated with GBS. The first epidemiological association was found in 1976, when an increased GBS risk was observed among persons who received the swine flu vaccine.^6^ Subsequently, an increased GBS risk was found after recombinant zoster vaccination.^7^ Associations between seasonal influenza vaccination and GBS have been inconsistently noted; some studies have shown a small increase in GBS risk among vaccinated persons while others have not.^8,9^

Postauthorization and postlicensure monitoring of adverse events are essential components of the US COVID-19 vaccination program.^10^ Initial surveillance findings suggested that the Ad26.COV2.S (Janssen) vaccine, a replication-incompetent human adenovirus vector COVID-19 vaccine, was associated with increased risk of GBS, including an imbalance in spontaneous reports and GBS diagnoses for Ad26.COV2.S compared with mRNA COVID-19 vaccines (BNT162b2 [Pfizer-BioNTech] and mRNA-1273 [Moderna]).^11,12^ We described and compared reports to the Vaccine Adverse Events Reporting System (VAERS) of verified GBS after the 3 available US COVID-19 vaccines among people ages 18 years and older and investigated whether surveillance data supported an association between Ad26.COV2.S, BNT162b2, or mRNA-1273 vaccines and GBS within 21 and 42 days after vaccination.

## Methods

The Centers for Disease Control and Prevention (CDC) determined that this cohort study was part of public health surveillance (45 CFR §46.102[l][2]^13^) and did not require evaluation by an institutional review board or informed consent. This cohort study and its results followed the Strengthening the Reporting of Observational Studies in Epidemiology (STROBE) reporting guideline.

### Setting, Population, and Incidence Definition

VAERS is a national passive vaccine safety surveillance system that is coadministered by the CDC and Food and Drug Administration. The system accepts reports of adverse events after vaccination from vaccine recipients or their parents or guardians, clinicians, health care institutions, vaccine manufacturers, and members of the public regardless of whether the reported events could plausibly be associated with vaccination.^14^ VAERS reports include demographic information and medical history of the vaccinated person, type of vaccine or vaccines received, date of vaccination, possible adverse events experienced by the vaccinated person and date of onset, history of adverse events after vaccination, and current illnesses and medications.^14^ Each report is reviewed by a trained coder who assigns Medical Dictionary for Regulatory Activities (MedDRA) Preferred Terms (PTs)^15^ based on clinical information in the report and any additional information from medical records obtained through follow-up.

Among US (domestic) VAERS reports submitted from December 14, 2020, through January 28, 2022, for people ages 18 years and older who received a COVID-19 vaccine (Ad26.COV2.S, BNT162b2, or mRNA-1273), we searched for the following MedDRA PTs: acute motor axonal neuropathy, acute motor-sensory axonal neuropathy, autoimmune neuropathy, demyelinating polyneuropathy, demyelination, subacute inflammatory demyelinating polyneuropathy, immune mediated neuropathy, Guillain Barré syndrome, or Miller-Fisher syndrome. Reports with any of these MedDRA PTs were deemed possible GBS cases regardless of time to symptom onset after vaccination. Available medical records, including death certificates, were reviewed. If VAERS reports did not include medical records, we contacted clinicians or health care institutions and requested these records. Medical records were considered unavailable for review if we did not receive a response from a clinician or health care institution by April 15, 2022.

CDC staff collected clinical data from available records concerning GBS signs and symptoms (hyporeflexia or areflexia, paresthesia, ophthalmoplegia or ophthalmoparesis, ataxia, limb weakness, and corticospinal tract signs), GBS testing (cerebrospinal fluid analysis, nerve conduction studies, or electromyography), GBS treatment (intravenous immunoglobulin, plasmapheresis, or both), and whether a GBS diagnosis was made by a physician, particularly a neurologist. CDC clinical reviewers then determined whether GBS reports met the Brighton Collaboration case definition for GBS.^1^ Brighton Collaboration criteria are used to assign diagnostic certainty to GBS cases.^1^ A Brighton level 1 case corresponds to the highest level of GBS diagnostic certainty, while level 4 corresponds to suspected GBS cases.^1^ GBS cases classified as Brighton level 1, 2, or 3 were considered verified GBS for this analysis. We obtained data about self-reported patient demographic characteristics, clinical and medical history, COVID-19 vaccine type and vaccination date, history of flulike or gastrointestinal symptoms within 42 days of the most recent COVID-19 vaccination, and time to GBS symptom onset after vaccination. Race and ethnicity were self-reported. Individuals were classified as Hispanic if categorized as having Hispanic ethnicity regardless of whether race was known. Individuals who identified as non-Hispanic and more than 1 race were classified as having multiple races. Individuals were classified as having unknown race and ethnicity if both race and ethnicity were missing. Categories for ethnicity were Hispanic and non-Hispanic; categories for race were American Indian or Alaska Native, Asian, Black, Native Hawaiian or Pacific Islander, White, unknown, and multiple races. Race and ethnicity were evaluated to assess racial distribution of GBS cases after COVID-19 vaccination. Duplicate VAERS reports were consolidated into a single report. We obtained data about number of COVID-19 doses administered during the surveillance period among people ages 18 years and older from the COVID Data Tracker.^16^ Two secondary reviewers (T.R.M. and J.R.S.) examined a 20% random sample of verified GBS reports (60 of 295 [20.3%]) to provide a quality check on the initial adjudication of verified GBS cases made by primary reviewers.

### Statistical Analysis

Descriptive statistics were used to describe verified GBS cases by type of COVID-19 vaccine received (Ad26.COV2.S, BNT162b2, or mRNA-1273). We calculated reporting rates (cases/1 000 000 vaccine doses administered) and reporting incidence rates (cases/100 000 person-y) of verified GBS cases with symptom onset within 21 and 42 days after vaccination by COVID-19 vaccine type. Person-time at risk was calculated using cumulative vaccine administration data. For person-time at risk (in person-y) during 21-day and 42-day postvaccination intervals, the calculations were N × 21/365.25 and N × 42/365.25, respectively, where N was number of vaccine doses administered. We selected a 21-day postvaccination risk interval because a second dose of mRNA COVID-19 vaccine could be received after this interval. The 42-day risk interval is commonly used in vaccine safety surveillance studies for GBS because higher GBS rates were observed within 5 to 6 weeks (35-42 days) of swine flu vaccination during the 1976 national swine flu immunization program.^6^ To estimate whether GBS reporting rates after Ad26.COV2.S vaccination were significantly different from rates after BNT162b2 or mRNA-1273 vaccination within the 21-day and 42-day postvaccination intervals, we calculated unadjusted reporting rate ratios (RRRs) (ie, GBS reporting rate after Ad26.COV2.S divided by reporting rate after BNT162b2 or mRNA-1273, respectively) using Poisson regression models.

We performed observed-to-expected (OE) analyses using all verified GBS cases and after stratifying by sex (male and female) and age group (18-49 years, 50-64 years, and ≥65 years), for each COVID-19 vaccine. We compared the observed number of verified GBS cases among vaccinated persons to an expected number of GBS cases in the general population during 21-day and 42-day risk intervals using pre-COVID-19 published GBS background rates.^17^ We derived GBS background rates based on the work of Sejvar et al,^3^ who estimated GBS background rates as a function of age group using a formula: exp[−12.0771 + 0.01813 (age in years)] × 100 000, where *exp* is the exponential function and age is the midpoint of the selected age group.^3^ For example, in the ages 18 to 29 years group, the midpoint is 24 years; thus, the GBS background rate is estimated as 0.88/100 000 person-years. We then used age-specific background rates to estimate an expected number of GBS reports for 21- and 42-day postvaccination risk intervals.^17^ We derived age-specific GBS background rates for males using the formula exp[−12.4038 + 0.01914(age in years) + 0.5777] × 100 000 and for females using the formula exp[−12.4038 + 0.01914(age in years)] × 100 000.^3^ Each respective background rate was used to estimate an expected number of GBS cases by age group and by sex for the 2 postvaccination risk intervals.^17^

OE ratios after Ad26.COV2.S, BNT162b2, or mRNA-1273 vaccination for 21-day and 42-day intervals were estimated by dividing the number of observed cases among doses administered by the expected number of cases based on historical background rates; 95% CIs were modeled using the Poisson distribution.^18^ *P* values were 2-sided, and *P* values < .05 were considered statistically significant. Analyses were done using SAS statistical software version 9.4 (SAS Institute).

## Results

During the surveillance period from December 14, 2020, through January 28, 2022, a total of 487 651 785 COVID-19 vaccine doses were administered in the US (17 944 515 Ad26.COV2.S doses [3.7%], 266 859 784 BNT162b2 doses [54.7%], and 202 847 486 mRNA-1273 doses [41.6%]). Using the MedDRA search strategy, we identified 912 possible GBS reports in VAERS; 806 of these reports (88.4%) had medical records available for review. After review and adjudication of these 806 VAERS reports and associated medical records, we verified 295 reports of individuals with GBS (12 Asian [4.1%], 18 Black [6.1%], and 193 White [65.4%]; 17 Hispanic [5.8%]; 169 males [57.3%]; median [IQR] age, 59 [46-68] years) per the Brighton Collaboration GBS case definition. These included 72 level 1 cases, 83 level 2 cases, 3 level 2 Miller-Fisher syndrome cases, 36 level 3 cases, and 1 level 3 Miller-Fisher syndrome case. Among verified cases, 82 cases listed Ad26.COV2.S vaccination (27.8%), 104 listed BNT162b2 vaccination (35.3%), 107 listed mRNA-1273 vaccination (36.3%), and 2 listed receipt of an unknown COVID-19 vaccine (0.6%) (Table 1).^3,17^ Among verified reports, 275 documented hospitalization (93.2%).

**Table 1.** Characteristics of Verified of GBS Cases

Characteristic	Reported individuals with GBS, No. (%)				
	Ad26.COV2.S (n = 82 [27.8%])	BNT162b2 (n = 104 [35.3%])	mRNA-1273 (n = 107 [36.3%])	Unknown vaccine (n = 2 [0.6%])	Any vaccine (N = 295)
Race and ethnicity^a^					
Hispanic	6 (7.3)	4 (3.9)	7 (6.5)	0	17 (5.8)
Non-Hispanic					
American Indian or Alaska Native	2 (2.4)	0	2 (1.9)	0	4 (1.4)
Asian	1 (1.2)	7 (6.7)	4 (3.7)	0	12 (4.1)
Black	4 (4.9)	6 (5.8)	8 (7.5)	0	18 (6.1)
Native Hawaiian or Pacific Islander	0	0	0	0	0
White	57 (69.5)	71 (68.3)	64 (59.8)	1 (50.0)	193 (65.4)
Unknown	12 (14.6)	14 (13.5)	19 (17.8)	1 (50.0)	46 (15.6)
Multiple races	0	2 (1.9)	3 (2.8)	0	5 (1.7)
Sex					
Male	45 (54.9)	62 (59.6)	60 (56.1)	2 (100.0)	169 (57.3)
Female	37 (45.1)	42 (40.4)	47 (43.9)	0	126 (42.7)
Hospitalization	80 (97.6)	96 (92.3)	97 (90.7)	2 (100.0)	275 (93.2)
Death	2 (2.4)	4 (3.8)	4 (3.7)	0	10 (3.3)
Age, median (IQR) [range]	57.0 (50.0-63.0) [22.0-80.0]	57.5 (40.0-68.0) [20.0-90.0]	63.0 (51.0-72.0) [19.0-87.0]	53.5 (47.0-60.0) [47.0-60.0]	59.0 (46.0-68.0) [19.0-90.0]
Cases 21 d after vaccination, No.	59	77	72	1	209
Time from vaccination to symptom onset, median (IQR), d	11.0 (6.0-14.0)	7.0 (2.0-12.0)	7.5 (2.5-12.5)	12.0 (12.0-12.0)	8.0 (3.0-13.0)
Cases 42 d after vaccination	73	90	89	1	253
Time from vaccination to symptom onset, median (IQR), d	13.0 (7.0-19.0)	7.0 (3.0-14.0)	10.0 (4.0-18.0)	12.0 (12.0-12.0)	10.0 (5.0-17.0)
Type of reporter					
Clinician or health care institution	53 (64.6)	58 (55.8)	73 (68.2)	1 (50.0)	185 (62.7)
Patient	10 (12.2)	22 (21.1)	16 (14.9)	0	48 (16.3)
Manufacturer	6 (7.3)	8 (7.7)	2 (1.9)	0	16 (5.4)
Parent, guardian, or caregiver	2 (2.4)	3 (2.9)	1 (0.9)	0	6 (2.0)
Other	11 (3.4)	13 (12.5)	15 (14.0)	1 (50.0)	40 (13.6)
Brighton level					
1	25 (30.5)	26 (25.0)	20 (18.7)	1 (50.0)	72 (24.4)
2	48 (58.5)	67 (64.4)	68 (63.6)	0	183 (62.0)
2 Miller-Fisher syndrome	1 (1.2)	0	2 (1.9)	0	3 (1.0)
3	8 (9.8)	11 (10.6)	16 (14.9)	1 (50.0)	36 (12.2)
3 Miller-Fisher syndrome	0	0	1 (0.9)	0	1 (0.4)

Abbreviation: GBS, Guillain-Barré syndrome.

^a^ Race and ethnicity were self-reported. Individuals were classified as Hispanic if categorized as having Hispanic ethnicity regardless of whether race was known. Individuals who identified as non-Hispanic and more than 1 race were classified as having multiple races. Individuals were classified as having unknown race and ethnicity if both race and ethnicity were missing.

Within 21 days after vaccination, 209 reports of GBS documented symptoms (70.8%); 253 GBS reports documented symptoms within 42 days after vaccination (85.8%). Of 209 GBS cases that occurred within 21 days of vaccination, 59 cases (28.2%) occurred after Ad26.COV2.S, 77 cases (36.8%) occurred after BNT162b2, and 72 cases (34.4%) occurred after mRNA-1273 vaccination. The type of COVID-19 vaccine received was unknown in 1 case. The median (IQR) time from vaccination to symptom onset among GBS cases occurring within 21 days after vaccination was 8.0 (3.0-13.0) days (11.0 [6.0-14.0] days after Ad26.COV2.S, 7.0 [2.0-12.0] days after BNT162b2, and 7.5 [2.5-12.5] days after mRNA-1273 vaccination). Of 253 GBS cases that occurred within 42 days of vaccination, 73 cases (28.9%) occurred after Ad26.COV2.S, 90 cases (35.6%) occurred after BNT162b2, and 89 cases (35.2%) occurred after mRNA-1273 vaccination. The type of COVID-19 vaccine received was unknown in 1 case. The median (IQR) time from vaccination to symptom onset among GBS cases occurring within 42 days after vaccination was 10.0 (5.0-17.0) days (13.0 [7.0-19.0] days after Ad26.COV2.S, 7.0 [3.0-14.0] days after BNT162b2, and 10.0 [4.0-18.0] days after mRNA-1273 vaccination). The Figure shows a distribution of verified GBS cases by time from vaccination to symptom onset.

**Figure.**
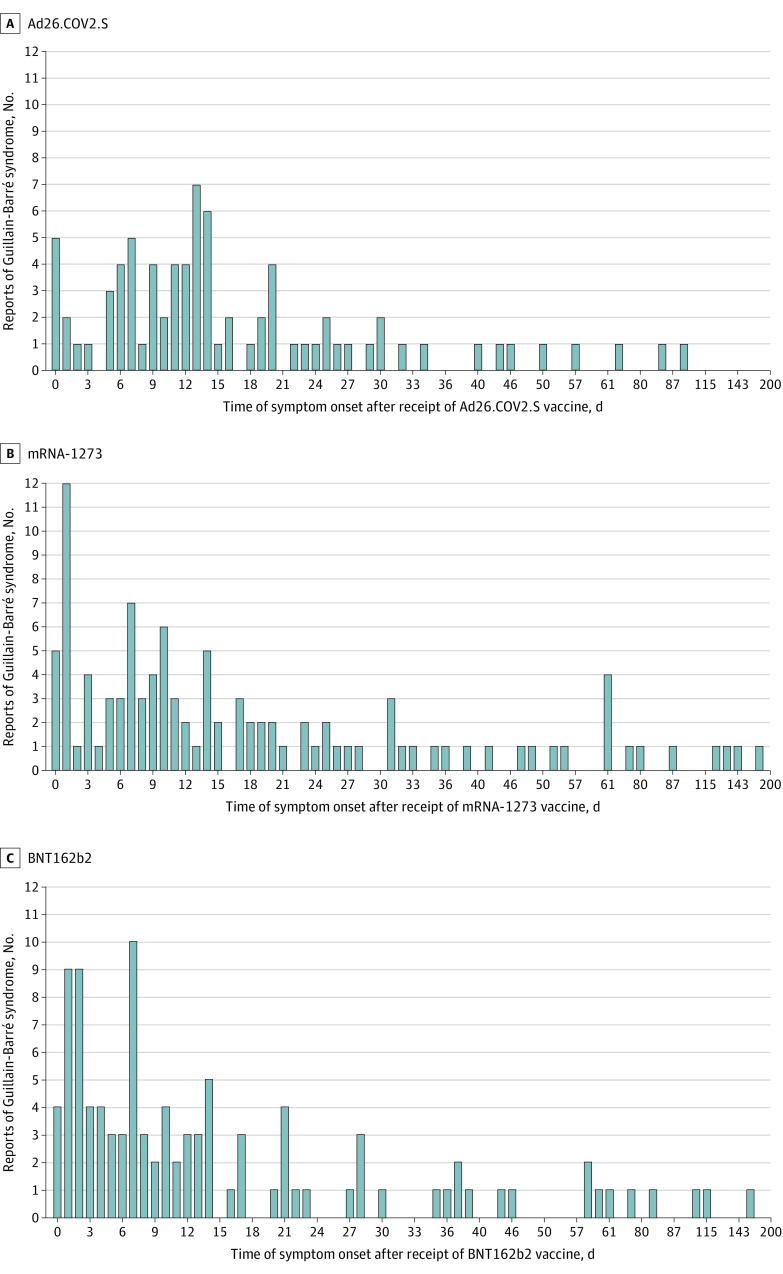
Distribution of Guillain-Barré Syndrome by COVID-19 Vaccine

There were 10 deaths reported (2 deaths after Ad26.COV2.S, 4 after BNT162b2, and 4 after mRNA-1273 vaccination) among all verified GBS cases. Demographic characteristics and clinical summaries of each individual who died are shown in the eTable in Supplement 1. Most individuals who died were non-Hispanic White (8 individuals [80.0%]) and male (7 males [70.0%]), received mechanical ventilation (7 individuals) and intravenous immunoglobulin treatment (10 individuals [100%]), and were categorized as Brighton level 2 (6 individuals [60.0%]); the median (range) age was 70 (57-88) years. The median (IQR) time from vaccination to symptom onset was 17.5 (5.0-70.0) days. GBS was determined to be the cause of death in 7 reports (70.0%) after review of death certificates (5 reports) and clinical notes (5 reports) by 2 clinician reviewers (J.R.S. and D.K.S.).

GBS reporting rates within 21 days after vaccination were 3.29 cases per 1 000 000 Ad26.COV2.S vaccine doses, 0.29 cases per 1 000 000 BNT162b2 vaccine doses, and 0.35 cases per 1 000 000 mRNA-1273 vaccine doses among all adults (Table 2). Stratifying by age group (18-49 years, 50-64 years, and ≥65 years), GBS reporting rates remained consistently highest after Ad26.COV2.S vaccination among all age groups within the 21-day postvaccination interval. GBS reporting rates within the 42-day postvaccination interval were also highest after Ad26.COV2.S vaccination among all adults (Ad26.COV2.S: 4.07 cases/1 000 000 doses; BNT162b2: 0.34 cases/1 000 000 doses; mRNA-1273: 0.44 cases/1 000 000 doses) and among all age groups.

**Table 2.** Rates of Verified GBS by Vaccine Type

Vaccine	No.		GBS reporting rate, No. cases/1 000 000 doses	Person-y, No.	Reporting incidence rate, No. cases/100 000 person-y	GBS RRR for Ad26.COV2.S vs other vaccines (95% CI)^a^
	GBS	Vaccine doses				
**21-d Postvaccination risk interval**						
Ages ≥18 y						
Ad26.COV2.S	59	17 944 515	3.29	1 031 717.49	5.72	NA
BNT162b2	77	266 859 784	0.29	15 343 067.66	0.50	11.40 (8.11-15.99)
mRNA-1273	72	202 847 486	0.35	11 662 689.13	0.62	9.26 (6.57-13.07)
Ages 18-49 y						
Ad26.COV2.S	13	9 735 496	1.34	559 741.04	2.32	NA
BNT162b2	33	132 439 289	0.25	7 614 579.24	0.43	5.36 (2.82-10.18)
mRNA-1273	14	83 064 308	0.17	4 775 771.30	0.29	7.92 (3.72-16.85)
Ages 50-64 y						
Ad26.COV2.S	36	5 482 229	6.57	315 200.02	11.42	NA
BNT162b2	16	68 858 186	0.23	3 958 992.21	0.40	28.26 (15.68-50.93)
mRNA-1273	23	54 462 150	0.42	3 131 294.05	0.73	15.55 (9.22-26.23)
Ages ≥65 y						
Ad26.COV2.S	10	2 726 790	3.67	156 776.43	6.38	NA
BNT162b2	28	65 562 309	0.43	3 769 496.21	0.74	8.56 (4.17-17.68)
mRNA-1273	35	65 321 028	0.54	3 755 623.79	0.93	6.84 (3.39-13.82)
**42-d Postvaccination risk interval**						
Ages ≥18 y						
Ad26.COV2.S	73	17 944 515	4.07	2 063 434.99	3.54	NA
BNT162b2	90	266 859 784	0.34	30 686 135.33	0.29	12.06 (8.86-16.43)
mRNA-1273	89	202 847 486	0.44	23 325 378.27	0.38	9.27 (6.80-12.63)
Ages 18-49 y						
Ad26.COV2.S	15	9 735 496	1.54	1 119 482.07	1.34	NA
BNT162b2	37	132 439 289	0.28	15 229 158.49	0.24	5.51 (3.03-10.04)
mRNA-1273	17	83 064 308	0.20	9 551 542.60	0.18	7.53 (3.76-15.07)
Ages 50-64 y						
Ad26.COV2.S	45	5 482 229	8.21	630 400.05	7.14	NA
BNT162b2	19	68 858 186	0.28	7 917 984.43	0.24	29.74 (17.4-50.86)
mRNA-1273	29	54 462 150	0.53	6 262 588.09	0.46	15.41 (9.67-24.58)
Ages ≥65 y						
Ad26.COV2.S	13	2 726 790	4.77	313 552.85	4.15	NA
BNT162b2	34	65 562 309	0.52	7 538 992.41	0.45	9.19 (4.85-17.42)
mRNA-1273	43	65 321 028	0.66	7 511 247.57	0.57	7.24 (3.89-13.47)

Abbreviations: GBS, Guillain-Barré syndrome; NA, not applicable.

^a^ Comparison is Ad26.COV2.S vs BNT162b2 and mRNA-1273 where reference is BNT162b2 and mRNA-1273, respectively.

We estimated RRRs to investigate whether GBS reporting varied by vaccine product received (Table 2). Within a 21-day postvaccination interval, GBS reporting rates among adults were greater after Ad26.COV2.S than BNT162b2 (RRR = 11.40; 95% CI, 8.11-15.99) or mRNA-1273 (RRR = 9.26; 95% CI, 6.57-13.07) vaccination. These same differences were also noted uniformly after stratifying by age group. Within a 42-day postvaccination interval, GBS reporting rates were greater after Ad26.COV2.S than BNT162b2 (RRR = 12.06; 95% CI, 8.86-16.43) and mRNA-1273 (RRR = 9.27; 95% CI, 6.80-12.63) vaccination among all adults and age groups when stratified by age group.

Significant differences were found in OE analysis of GBS cases within 21 days (OE ratio = 3.79; 95% CI, 2.88-4.88) and 42 days (OE ratio = 2.34; 95% CI, 1.83-2.94) after Ad26.COV2.S vaccination among all adults and individually by age group (Table 3).^3,17^ Similar patterns were found in sex-specific analyses. The OE ratios within 21 days of Ad26.COV2.S vaccination (males: 3.15; 95% CI, 2.23-4.37; females: 4.23; 95% CI, 2.65-6.39]) and within 42 days of Ad26.COV2.S vaccination (males: 1.84; 95% CI, 1.33-2.48; females: 2.89; 95% CI, 1.94-4.11]) were significantly increased. OE ratios for the 21-day postvaccination interval for BNT162b2 (0.33; 95% CI, 0.26-0.42) and mRNA-1273 (0.41; 95% CI, 0.32-0.51) and 42-day postvaccination interval for BNT162b2 (0.41; 95% CI, 0.32-0.51) and mRNA-1273 (0.25; 95% CI, 0.20-0.31) did not demonstrate significant differences between observed and expected numbers of GBS cases among all adults or after stratifying by age and sex (Table 4).^3,17^

**Table 3.** OE Analysis of Verified GBS After Ad26.COV2.S Vaccine

Variable	Observed GBS cases, No.	Vaccine doses, No.	Person-y, No.	Reporting incidence rate, No. cases/100 000 person-y	Background rate, No. cases/100 000 person-y^a^	Expected GBS cases, No.^b^	OE ratio (95% CI)
**Onset within 21 d after vaccination**							
Age, y							
All ≥18^c^	59	17 944 515	1 031 717.49	5.72	1.51	15.58	3.79 (2.88-4.88)
18-64	49	15 217 725	874 941.07	5.60	1.22	10.67	4.59 (3.40-6.07)
18-49	13	9 735 496	559 741.04	2.32	1.05	5.88	2.21 (1.18-3.78)
50-64	36	5 482 229	315 200.02	11.42	1.60	5.04	7.14 (5.00-9.89)
≥65	10	2 726 790	156 776.42	6.38	2.40	3.76	2.66 (1.27-4.89)
Sex by age, y							
Males							
All ≥18	37	9 859 009	566 842.40	6.53	2.06	11.68	3.15 (2.23-4.37)
18-64	32	8 562 989	492 327.90	6.50	1.63	8.02	3.98 (2.73-5.63)
18-49	7	5 621 981	323 235.05	2.17	1.40	4.53	1.55 (0.62-3.18)
50-64	25	2 941 008	169 092.86	14.78	2.18	3.69	6.75 (4.38-10.0)
≥65	5	1 296 020	74 514.49	6.71	3.19	2.38	2.10 (0.68-4.90)
Females							
All ≥18	22	7 880 034	453 061.50	4.86	1.15	5.21	4.23 (2.65-6.39)
18-64	17	6 470 127	371 999.08	4.57	0.92	3.42	4.96 (2.89-7.96)
18-49	6	3 991 277	229 477.94	2.61	0.79	1.81	3.31 (1.21-7.22)
50-64	11	2 478 850	142 521.14	7.72	1.22	1.74	6.34 (3.15-11.31)
≥65	5	1 409 907	81 062.41	6.17	1.79	1.45	3.45 (1.11-8.05)
**Onset within 42 d after vaccination**							
Age, y							
All ≥18^c^	73	17 944 515	2 063 434.99	3.54	1.51	31.20	2.34 (1.83-2.94)
18-64	60	15 217 725	1 749 882.13	3.43	1.22	21.34	2.81 (2.15-3.62)
18-49	15	9 735 496	1 119 482.09	1.34	1.05	11.8	1.28 (0.71-2.11)
50-64	45	5 482 229	630 400.05	7.14	1.6	10.10	4.46 (3.25-5.96)
≥65	13	2 726 790	313 552.85	4.14	2.4	7.53	1.73 (0.92-2.95)
Sex by age, y							
Males							
All ≥18	43	9 859 009	1 133 684.81	3.80	2.06	23.35	1.84 (1.33-2.48)
18-64	37	8 562 989	984 655.81	3.76	1.63	16.05	2.31 (1.62-3.18)
18-49	8	5 621 981	646 470.09	1.24	1.40	9.05	0.88 (0.38-1.74)
50-64	29	2 941 008	338 185.72	8.58	2.18	7.37	3.93 (2.63-5.65)
≥65	6	1 296 020	149 028.99	4.03	3.19	4.75	1.26 (0.46-2.75)
Females							
All ≥18	30	7 880 034	906 123.00	3.31	1.15	10.42	2.89 (1.94-4.11)
18-64	23	6 470 127	743 998.18	3.09	0.92	6.84	3.36 (2.13-5.05)
18-49	7	3 991 277	458 955.88	1.53	0.79	3.63	1.93 (0.77-3.97)
50-64	16	2 478 850	285 042.29	5.61	1.22	3.48	4.61 (2.63-7.47)
≥65	7	1 409 907	162 124.83	4.31	1.79	2.90	2.41 (0.97-4.97)

Abbreviations: GBS, Guillain-Barré Syndrome; OE, observed to expected.

^a^ Calculated based on Sejvar et al.^3^

^b^ Calculated as the product of daily incidence rate per person, number of vaccine doses, and the associated postvaccination time interval (21 days and 42 days).^17^

^c^ COVID-19 vaccine doses administered to males and females may not sum to vaccine doses administered to all adults because of missing sex responses for 205 472 Ad26.COV2.S vaccine doses.

**Table 4.** OE Analysis of Verified GBS After mRNA COVID-19 Vaccines

Variable	Observed GBS cases, No.	Vaccine doses, No.	Person-y, No.	Reporting incidence rate, No. cases/100 000 person-y	Background rate, No. cases/100 000 person-y^a^	Expected GBS cases, No.^b^	OE ratio (95% CI)
**Onset within 21 d after BNT162b2 vaccine**							
Age, y							
All ≥18^c^	77	266 859 784	15 343 067.66	0.50	1.51	231.68	0.33 (0.26-0.42)
18-64	49	201 297 475	11 573 571.46	0.42	1.22	141.20	0.35 (0.26-0.46)
18-49	33	132 439 289	7 614 579.24	0.43	1.05	79.95	0.41 (0.28-0.58)
50-64	16	68 858 186	3 958 992.21	0.40	1.60	63.34	0.25 (0.14-0.41)
≥65	28	65 562 309	3 769 496.21	0.74	2.30	86.70	0.32 (0.21-0.47)
Sex by age, y							
Males							
All ≥18	48	122 144 623	7 022 688.79	0.68	2.06	144.33	0.33 (0.25-0.44)
18-64	29	93 101 496	5 352 858.09	0.54	1.63	87.44	0.33 (0.22-0.48)
18-49	18	61 132 988	3 514 832.99	0.51	1.40	49.26	0.37 (0.22-0.58)
50-64	11	31 968 508	1 838 025.10	0.60	2.18	40.10	0.27 (0.14-0.49)
≥65	19	29 043 127	1 669 830.71	1.14	3.19	53.30	0.36 (0.21-0.56)
Females							
All ≥18	29	142 987 679	8 221 057.52	0.35	1.15	94.54	0.31 (0.21-0.44)
18-64	20	106 719 401	6 135 817.72	0.33	0.92	56.45	0.35 (0.22-0.55)
18-49	15	70 332 820	4 043 776.10	0.37	0.79	31.95	0.47 (0.26-0.77)
50-64	5	36 386 581	2 092 041.62	0.24	1.22	45.48	0.11 (0.04-0.26)
≥65	9	36 268 278	2 085 239.80	0.43	1.79	73.56	0.12 (0.06-0.23)
**Onset within 42 d after BNT162b2 vaccine**							
Age, y							
All ≥18^c^	90	266 859 784	15 343 067.66	0.59	1.51	463.36	0.19 (0.16-0.24)
18-64	56	201 297 475	11 573 571.46	0.48	1.22	282.40	0.20 (0.15-0.26)
18-49	37	132 439 289	7 614 579.24	0.49	1.05	159.91	0.23 (0.16-0.32)
50-64	19	68 858 186	3 958 992.21	0.48	1.60	126.69	0.15 (0.09-0.23)
≥65	34	65 562 309	3 769 496.20	0.90	2.30	173.40	0.20 (0.17-0.27)
Sex by age, y							
Males							
All ≥18	53	122 144 623	7 022 688.79	0.75	2.06	288.66	0.18 (0.14-0.24)
18-64	32	93 101 496	5 352 858.08	0.59	1.63	174.87	0.18 (0.13-0.26)
18-49	20	61 132 988	3 514 832.98	0.57	1.40	98.52	0.20 (0.12-0.31)
50-64	12	31 968 508	1 838 025.10	0.65	2.18	80.02	0.15 (0.08-0.26)
≥65	21	29 043 127	1 669 830.71	1.26	3.19	106.60	0.20 (0.12-0.30)
Females							
All ≥18	37	142 987 679	8 221 057.52	0.45	1.15	189.08	0.20 (0.14-0.27)
18-64	24	106 719 401	6 135 817.71	0.39	0.92	112.90	0.21 (0.14-0.32)
18-49	17	70 332 820	4 043 776.09	0.42	0.79	63.89	0.27 (0.15-0.43)
50-64	7	36 386 581	2 092 041.61	0.33	1.22	51.05	0.14 (0.05-0.28)
≥65	13	36 268 278	2 085 239.80	0.62	1.79	74.65	0.17 (0.09-0.30)
**Onset within 21 d after mRNA-1273 vaccine**							
Age, y							
All ≥18	72	202 847 486	11 662 689.13	0.62	1.51	176.11	0.41 (0.32-0.51)
18-64	37	137 526 458	7 907 065.34	0.47	1.22	96.47	0.38 (0.27-0.53)
18-49	14	83 064 308	4 775 771.30	0.29	1.05	50.15	0.28 (0.15-0.47)
50-64	23	54 462 150	3 131 294.05	0.73	1.60	50.10	0.46 (0.29-0.69)
≥65	35	65 321 028	3 755 623.78	0.93	2.30	86.70	0.40 (0.28-0.56)
Sex by age, y							
Males							
All ≥18	39	94 368 165	5 425 685.05	0.72	2.06	111.51	0.35 (0.25-0.48)
18-64	21	64 588 146	37 134 86.83	0.57	1.63	60.66	0.35 (0.21-0.53)
18-49	7	39 097 830	2 247 924.52	0.31	1.40	31.51	0.22 (0.09-0.46)
50-64	14	25 490 316	1 465 562.31	0.96	2.18	31.90	0.44 (0.24-0.74)
≥65	18	29 780 019	1 712 198.21	1.05	3.19	54.65	0.33 (0.20-0.52)
Females							
All ≥18	33	106 824 031	6 141 833.41	0.54	1.15	70.63	0.47 (0.32-0.66)
18-64	16	71 620 442	4 117 807.75	0.39	0.92	37.88	0.42 (0.24-0.69)
18-49	7	43 174 944	2 482 337.64	0.28	0.79	19.61	0.36 (0.14-0.74)
50-64	9	28 445 498	1 635 470.11	0.55	1.22	19.95	0.45 (0.21-0.86)
≥65	17	35 203 589	2 024 025.65	0.84	1.79	36.23	0.47 (0.27-0.75)
**Onset within 42 d after mRNA-1273 vaccine**							
Age, y							
All ≥18	89	202 847 486	11 662 689.13	0.76	1.51	352.21	0.25 (0.20-0.31)
18-64	46	137 526 458	7 907 065.35	0.58	1.22	192.93	0.24 (0.17-0.32)
18-49	17	83 064 308	4 775 771.30	0.36	1.05	100.29	0.17 (0.10-0.27)
50-64	29	54 462 150	3 131 294.05	0.93	1.60	100.20	0.29 (0.19-0.42)
≥65	43	65 321 028	3 755 623.78	1.14	2.30	173.40	0.25 (0.18-0.33)
Sex by age, y							
Males							
All ≥18	48	94 368 165	5 425 685.05	0.88	2.06	223.02	0.22 (0.16-0.29)
18-64	25	64 588 146	3 713 486.83	0.67	1.63	121.32	0.21 (0.13-0.30)
18-49	7	39 097 830	2 247 924.52	0.31	1.40	63.01	0.11 (0.04-0.23)
50-64	18	25 490 316	1 465 562.32	1.22	2.18	63.80	0.28 (0.17-0.45)
≥65	23	29 780 019	1 712 198.22	1.34	3.19	109.30	0.21 (0.13-0.32)
Females							
All ≥18	41	106 824 031	6 141 833.41	0.67	1.15	141.26	0.29 (0.21-0.39)
18-64	21	71 620 442	4 117 807.75	0.51	0.92	75.77	0.28 (0.17-0.42)
18-49	10	43 174 944	2 482 337.64	0.40	0.79	39.22	0.25 (0.12-0.47)
50-64	11	28 445 498	1 635 470.11	0.67	1.22	39.91	0.28 (0.14-0.49)
≥65	20	35 203 589	2 024 025.65	0.98	1.79	72.46	0.28 (0.17-0.43)

^a^ Calculated based on Sejvar et al.^3^

^b^ Calculated as the product of daily incidence rate per person, number of vaccine doses, and the associated postvaccination time interval (21 days and 42 days).^17^

^c^ COVID-19 vaccine doses administered to males and females may not sum to vaccine doses administered to all adults because of missing sex responses for 1 727 482 BNT162b2 vaccine doses and 1 655 290 mRNA-1273 vaccine doses.

## Discussion

In this retrospective cohort study, we identified 295 verified GBS cases among VAERS reports submitted from December 2020 through January 2022. GBS reporting after Ad26.COV2.S vaccination was approximately 9 to 12 times more common than after BNT162b2 or mRNA-1273 vaccination within 21- and 42-day postvaccination intervals. Similarly, observed GBS cases after Ad26.COV2.S vaccination were 2 to 3 times greater than expected based on background rates within 21- and 42-day postvaccination intervals. There was no significant difference between observed and expected numbers of GBS cases after either mRNA COVID-19 vaccine.

Our findings are similar to those from 2 previous studies. An analysis of VAERS reports made during February to July 2021 showed a potential association between Ad26.COV2.S vaccination and presumptive (ie, unverified) GBS cases.^11^ Our analysis included reports of verified GBS cases submitted from December 2020 through January 2022 that met levels 1 through 3 of the Brighton Collaboration GBS case definition. We compared the associations of Ad26.COV2.S, BNT162b2, and mRNA-1273 vaccination with GBS. An analysis of surveillance data collected during December 2020 to November 2021 by the Vaccine Safety Datalink (VSD) showed an imbalance between verified GBS diagnoses after Ad26.COV2.S vaccination compared with mRNA COVID-19 vaccination.^12^ Our US-level analysis with VAERS data included verified cases from health care settings not necessarily included in VSD. Our data covered a longer surveillance period than the previous VAERS analysis of presumptive GBS cases. Our results include almost 300 reports of verified GBS cases in VAERS over a 14-month period and therefore may provide a more precise estimate of the relative risk of GBS after Ad26.COV2.S vaccination.

Of 10 deaths reported among individuals aged 57 through 88 years, GBS was the documented cause of death after medical record and death certificate review for 7 individuals. Increasing age is associated with a poorer GBS prognosis and an increased mortality risk.^19^ Of these deaths, 8 occurred after mRNA COVID-19 vaccination; however, there was no epidemiologic evidence to suggest an association between either mRNA vaccine and GBS. There were 2 deaths reported after Ad26.COV2.S vaccination. In 1 death, the individual was noted to have onset of GBS symptoms at 70 days after vaccination, which is outside an epidemiologically accepted risk interval to assume an association between vaccination and GBS. The other individual was noted to have symptom onset 5 days after Ad26.COV2.S vaccination; GBS was documented as the cause of death. Based on the available evidence, it is biologically plausible that Ad26.COV2.S vaccination may have been associated with the death, although definitively establishing such an association is difficult with the available information and conclusions about causality cannot be made in this observational study. Under COVID-19 vaccine Emergency Use Authorization regulations, clinicians and health care institutions are required to report deaths and life-threatening events and other specified serious adverse events that occur after COVID-19 vaccination to VAERS regardless of the potential association of vaccination with these outcomes.^20^ These reporting requirements are different from those for other licensed and routinely recommended vaccines; therefore, it is difficult to make direct comparisons between numbers of deaths reported to VAERS after COVID-19 vaccination and deaths after vaccines routinely administered to older adults.^21^

The specific contributing cause of and risk factors associated with individual GBS cases are often unclear, and its pathogenesis remains incompletely understood. However, molecular mimicry may play a role in the etiology of GBS.^4^ Ad26.COV2.S is a recombinant vaccine that uses a nonreplicating adenovirus vector encoding the SARS-CoV-2 spike protein to trigger an immunologic antibody response.^22^ It is theoretically possible that antibodies induced by Ad26.COV2.S vaccine may cross-react with glycoproteins on the myelin sheath of the axons of peripheral nerves, resulting in GBS.^23^ An increased risk of GBS after ChAdOx1 nCov-19 COVID-19 vaccination (AstraZeneca) has also been reported.^24,25^ This vaccine uses a replication-incompetent chimpanzee adenovirus vector and has been widely administered in Europe.^24,25^ In 1 study, the number of GBS cases within 14 days of ChAdOx1 nCov-19 vaccination was 1.4- to 10-fold greater than expected.^24^ Increased risk of GBS after these vaccines may suggest an adenovirus vector vaccine class association, at least with respect to COVID-19 vaccines. This possibility merits further evaluation and research concerning plausible mechanisms.

### Limitations and Strengths

There are limitations to this analysis. First, VAERS data are subject to underreporting because VAERS is a passive surveillance system and reports received may be incomplete. While additional records were requested for review, they may not have been received at all or in a timely manner. Second, some cases that may have met the Brighton Collaboration case definition were likely missed among 106 possible cases that lacked medical records for review. Third, GBS background rates during the COVID-19 pandemic may be different than prepandemic rates; such differences could affect the interpretation of any risk of vaccine-associated GBS.^17,26^ Fourth, while we checked data quality and integrity, a secondary review was conducted for only 20% of verified GBS cases to confirm that they met the Brighton Collaboration case definition. Fifth, we were not able to conduct dose-specific analyses.

Strengths of this VAERS analysis include the use of a specific, consistently applied definition of GBS among reports received from the entire US vaccinated population. Another strength of our analysis was our ability to conduct analyses of verified GBS cases by age group and sex, unlike other studies.

## Conclusions

In this retrospective cohort study, we found evidence for increased risks of GBS within 21- and 42-day intervals after Ad26.COV2.S vaccination. The absolute risk of GBS after Ad26.COV2.S vaccination was likely on the order of several cases per million doses of vaccine administered. Conversely, we did not find increased risks for GBS after receipt of either mRNA COVID-19 vaccine, suggesting that GBS cases observed after mRNA COVID-19 vaccination may represent background GBS incidence. The Advisory Committee on Immunization Practices preferentially recommends that individuals aged 18 years and older receive an mRNA COVID-19 vaccine rather than the Ad26.COV2.S vaccine when both types of COVID-19 vaccine are available. Notably, this recommendation is based primarily on the increased risk of the rare serious condition thrombosis with thrombocytopenia syndrome after Ad26.COV2.S vaccination, but it is also based on a recognized association with GBS.^27^

## Notes

Supplement 1. **eTable.** Demographic Characteristics and Clinical Summary of 10 Individuals Who Died

Supplement 2.
Data Sharing Statement

## References

[1] Sejvar JJ, Kohl KS, Gidudu J, ; Brighton Collaboration GBS Working Group. Guillain-Barré syndrome and Fisher syndrome: case definitions and guidelines for collection, analysis, and presentation of immunization safety data. Vaccine. 2011;29(3):599-612. doi:10.1016/j.vaccine.2010.06.003 20600491

[2] Centers for Disease Control and Prevention. Guillain-Barré Syndrome and vaccines. Accessed May 21, 2022. https://www.cdc.gov/vaccinesafety/concerns/guillain-barre-syndrome.html

[3] Sejvar JJ, Baughman AL, Wise M, Morgan OW. Population incidence of Guillain-Barré syndrome: a systematic review and meta-analysis. Neuroepidemiology. 2011;36(2):123-133. doi:10.1159/000324710 21422765 PMC5703046

[4] van den Berg B, Walgaard C, Drenthen J, Fokke C, Jacobs BC, van Doorn PA. Guillain-Barré syndrome: pathogenesis, diagnosis, treatment and prognosis. Nat Rev Neurol. 2014;10(8):469-482. doi:10.1038/nrneurol.2014.121 25023340

[5] van Doorn PA, Ruts L, Jacobs BC. Clinical features, pathogenesis, and treatment of Guillain-Barré syndrome. Lancet Neurol. 2008;7(10):939-950. doi:10.1016/S1474-4422(08)70215-1 18848313

[6] Schonberger LB, Bregman DJ, Sullivan-Bolyai JZ, . Guillain-Barre syndrome following vaccination in the National Influenza Immunization Program, United States, 1976—1977. Am J Epidemiol. 1979;110(2):105-123. doi:10.1093/oxfordjournals.aje.a112795 463869

[7] Goud R, Lufkin B, Duffy J, . Risk of Guillain-Barré syndrome following recombinant zoster vaccine in Medicare beneficiaries. JAMA Intern Med. 2021;181(12):1623-1630. doi:10.1001/jamainternmed.2021.6227 34724025 PMC8561433

[8] Greene SK, Rett MD, Vellozzi C, . Guillain-Barré syndrome, influenza vaccination, and antecedent respiratory and gastrointestinal infections: a case-centered analysis in the Vaccine Safety Datalink, 2009–2011. PLoS One. 2013;8(6):e67185. doi:10.1371/journal.pone.0067185 23840621 PMC3694016

[9] Salmon DA, Proschan M, Forshee R, ; H1N1 GBS Meta-Analysis Working Group. Association between Guillain-Barré syndrome and influenza A (H1N1) 2009 monovalent inactivated vaccines in the USA: a meta-analysis. Lancet. 2013;381(9876):1461-1468. doi:10.1016/S0140-6736(12)62189-8 23498095

[10] Lee GM, Romero JR, Bell BP. Postapproval vaccine safety surveillance for COVID-19 vaccines in the US. JAMA. 2020;324(19):1937-1938. doi:10.1001/jama.2020.19692 33064152

[11] Woo EJ, Mba-Jonas A, Dimova RB, Alimchandani M, Zinderman CE, Nair N. Association of receipt of the Ad26.COV2.S COVID-19 vaccine with presumptive Guillain-Barré syndrome, February-July 2021. JAMA. 2021;326(16):1606-1613. doi:10.1001/jama.2021.16496 34617967 PMC8498927

[12] Hanson KE, Goddard K, Lewis N, . Incidence of Guillain-Barré syndrome after COVID-19 vaccination in the Vaccine Safety Datalink. JAMA Netw Open. 2022;5(4):e228879. doi:10.1001/jamanetworkopen.2022.8879 35471572 PMC9044108

[13] US Department of Health and Human Services. Protection of human subjects. 45 CFR §46. Revised January 19, 2017. Accessed December 28, 2020. https://www.hhs.gov/ohrp/regulations-and-policy/regulations/45-cfr-46/revised-common-rule-regulatory-text/index.html#46.102

[14] Centers for Disease Control and Prevention. Vaccine adverse event reporting system (VAERS). Accessed May 21, 2022. https://www.cdc.gov/vaccinesafety/ensuringsafety/monitoring/vaers/index.html

[15] International Council for Harmonisation of Technical Requirements for Pharmaceuticals for Human Use. Medical dictionary for regulatory activities. Accessed May 21, 2022. https://www.meddra.org/

[16] Centers for Disease Control and Prevention. COVID data tracker. Accessed May 21, 2022. https://covid.cdc.gov/covid-data-tracker/#vaccinations_vacc-total-admin-rate-total

[17] Abara WE, Gee J, Delorey M, . Expected rates of select adverse events after immunization for coronavirus disease 2019 vaccine safety monitoring. J Infect Dis. 2022;225(9):1569-1574. doi:10.1093/infdis/jiab628 34958099 PMC8755334

[18] Fleiss JL, Levin B, Paik MC. Statistical Methods for Rates and Proportions. 3rd ed. John Wiley & Sons; 2003. doi:10.1002/0471445428.

[19] Willison HJ, Jacobs BC, van Doorn PA. Guillain-Barré syndrome. Lancet. 2016;388(10045):717-727. doi:10.1016/S0140-6736(16)00339-1 26948435

[20] Centers for Disease Control and Prevention. Reporting adverse events following vaccination. Accessed May 21, 2022. https://www.cdc.gov/vaccinesafety/hcproviders/reportingadverseevents.html

[21] Moro PL, Arana J, Cano M, Lewis P, Shimabukuro TT. Deaths reported to the vaccine adverse event reporting system, United States, 1997–2013. Clin Infect Dis. 2015;61(6):980-987. doi:10.1093/cid/civ423 26021988 PMC6771280

[22] Sadoff J, Le Gars M, Shukarev G, . Interim results of a phase 1-2a trial of Ad26.COV2.S COVID-19 vaccine. N Engl J Med. 2021;384(19):1824-1835. doi:10.1056/NEJMoa2034201 33440088 PMC7821985

[23] Thant HL, Morgan R, Paese MM, Persaud T, Diaz J, Hurtado L. Guillain-Barré syndrome after Ad26.COV2.S vaccination. Am J Case Rep. 2022;23:e935275-e935271. doi:10.12659/AJCR.935275 35157644 PMC8855329

[24] Maramattom BV, Krishnan P, Paul R, . Guillain-Barré syndrome following ChAdOx1-S/nCoV-19 vaccine. Ann Neurol. 2021;90(2):312-314. doi:10.1002/ana.26143 34114256

[25] Allen CM, Ramsamy S, Tarr AW, . Guillain–Barré syndrome variant occurring after SARS-CoV-2 vaccination. Ann Neurol. 2021;90(2):315-318. doi:10.1002/ana.26144 34114269

[26] Black SB, Law B, Chen RT, . The critical role of background rates of possible adverse events in the assessment of COVID-19 vaccine safety. Vaccine. 2021;39(19):2712-2718. doi:10.1016/j.vaccine.2021.03.016 33846042 PMC7936550

[27] Centers for Disease Control and Prevention. Summary document for interim clinical considerations for use of COVID-19 vaccines currently authorized or approved in the United States. Accessed May 21, 2022. https://www.cdc.gov/vaccines/covid-19/downloads/summary-interim-clinical-considerations.pdf

