# Errors in Abstract, Results, and Discussion

**DOI:** 10.1001/jamanetworkopen.2023.15740

**Published:** 2023-05-12

In the Original Investigation titled “Reports of Guillain-Barré Syndrome After COVID-19 Vaccination in the United States,”^1^ published February 1, 2023, there were errors in the Results in the number of Brighton level 1 cases and the observed-to-expected (OE) ratio data for the BNT162b2 vaccine 42-day postvaccination interval. The needed terms “increase” or “increased” were missing in descriptions of OE ratios and risk of Guillain-Barré syndrome for BNT162b2 and mRNA-1273 vaccines in the Abstract, Results, and Discussion. This article has been corrected.^1^
