# Supplementary material for: Reports of Guillain-Barré Syndrome After COVID-19 Vaccination in the United States
Source: JAMA Netw Open. 2023 Feb 1;6(2):e2253845. doi: 10.1001/jamanetworkopen.2022.53845 (PMC9892957; doi:10.1001/jamanetworkopen.2022.53845)
Supplement: Supplement 1. — eTable. Demographic Characteristics and Clinical Summary of 10 Individuals Who Died [file jamanetwopen-e2253845-s001.pdf]

Supplemental Online Content

Abara WE, Gee J, Marquez P, et al. Reports of Guillain-Barré syndrome after COVID-19 vaccination in the United States. *JAMA Netw Open*. 2023;6(2):e2253845. doi:10.1001/jamanetworkopen.2022.53845

eTable. Demographic Characteristics and Clinical Summary of 10 Individuals Who Died

This supplemental material has been provided by the authors to give readers additional information about their work.

© 2023 Abara WE et al. *JAMA Network Open.*

eTable. Demographic Characteristics and Clinical Summary of 10 Individuals Who Died

Age group, years[a]	Gender	Symptom onset after vaccination	Dose	Brighton Level	Vaccine	Clinical summary	Death certificate	Cause of Death
55–59	Male	5	1	3	Ad.26.COV2.S (Janssen)	Presented with paresthesia and progressive upper and lower extremity weakness. Neurological examination: limb weakness, hyporeflexia, and decreased sensation. Lumbar puncture: CSF protein high (300mg/dl), WBC=5/µl. Diagnosis: GBS; Treatment: IV immunoglobulin. Patient experienced breathing difficulties, became hypoxic, and required mechanical ventilation. There is no information about circumstances surrounding death.	Yes	GBS
75–79	Male	5	2	1	mRNA-1273 (Moderna)	Presented with lower extremity weakness, numbness, and inability to stand. Diagnosed with COVID-19 8 months prior to presentation Neurological examination: areflexic globally, muscle weakness, dysphagia, and dysarthria. Lumbar puncture: elevated CSF protein and normal WBC. Electromyography results consistent with GBS. Diagnosis: GBS. Treatment: IV immunoglobulin. Patient experienced breathing difficulties, became hypoxic, and required mechanical ventilation. Clinical condition worsened and patient died from GBS complications per medical records.	No	GBS
70–74	Female	28	2	2	BNT162b2 (Pfizer)	Presented with upper and lower extremity weakness and progressive difficulty walking. Neurological examination: dysphagia, dysarthria, muscle weakness, and hyporeflexia. Lumbar puncture results consistent with GBS (results not provided) per medical records. Diagnosis: GBS. Treatment: IV immunoglobulin. Patient experienced breathing difficulties, became hypoxic, and required mechanical ventilation. Clinical condition worsened and patient died.	Yes	GBS
70–74	Male	7	1	2	mRNA-1272 (Moderna)	Presented with dizziness and lower extremity weakness. Comorbid cardiac conditions. Neurological examination: hyporeflexia and muscle weakness. Lumbar puncture: elevated CSF protein and normal WBC. Diagnosis: GBS; Treatment: IV immunoglobulin. Patient suffered cardiopulmonary arrest during hospital stay and was unable to be resuscitated per medical records.	No	Sudden cardio-pulmonary arrest
50–54	Male	60	2	2	mRNA-1273 (Moderna)	Presented with nausea, vomiting, lower extremity weakness and numbness. Comorbid coronary artery disease and diagnosed with COVID-19 8 months prior to presentation. Neurological examination: hyporeflexia and muscle weakness. Lumbar puncture: elevated CSF protein (76.2mg/dl) and normal WBC. Diagnosis: GBS; Treatment: IV immunoglobulin. Condition	Yes	Coronary artery disease

© 2023 Abara WE et al. *JAMA Network Open.*

						improved and patient was transferred to rehabilitation center where he collapsed and died during physical therapy.		
65–69	Male	7	1	2	BNT162b2 (Pfizer)	Presented with tingling and numbness in feet, difficulty standing and walking, paresthesia, and mild dysphagia. Neurological examination: upper and lower extremity muscle weakness, hyporeflexia. Lumbar puncture: results not provided. Diagnosis: GBS; Treatment: IV immunoglobulin and plasmapheresis. Patient experienced breathing difficulties, became hypoxic, and required mechanical ventilation. Clinical condition worsened and patient was transferred to long-term care facility where patient died. GBS was cause of death per medical records.	No	GBS
60–64	Male	92	2	1	mRNA-1273 (Moderna)	Presented with weakness in upper and lower extremities, numbness, and shortness of breath. Neurological examination: hyporeflexia and upper and lower extremity muscle weakness. Lumbar puncture: elevated protein, results not provided. Diagnosis: GBS; Treatment: IV immunoglobulin. Patient experienced breathing difficulties, became hypoxic, and required mechanical ventilation. Clinical condition worsened, comfort care provided, and patient died. GBS was cause of death per medical records.	No	GBS
85–89	Female	109	2	2	BNT162b2 (Pfizer)	Presented with quadriplegia. Neurological examination: hyporeflexia and upper and lower extremity muscle weakness. Lumbar puncture: elevated protein (88mg/dl). Diagnosis: GBS; Treatment: IV immunoglobulin and plasmapheresis. Patient experienced breathing difficulties, became hypoxic, and required mechanical ventilation. Clinical condition worsened and patient died.	Yes	GBS
85–89	Female	2	2	2	BNT162b2 (Pfizer)	Patient presented with lower extremity pain and weakness. Neurological examination: upper and lower extremity muscle weakness, hyporeflexia. Lumbar puncture: elevated protein and normal WBC (results not provided). Electromyography results consistent with axonal GBS. Diagnosis: GBS; Treatment: IV immunoglobulin and plasmapheresis. Patient transferred to nursing home where clinical condition continued to decline until death. There is no information about circumstances surrounding death in clinical records.	No	Unknown
65–69	Male	70	2	2	Ad.26.COV2.S (Janssen)	Presented with ascending paresthesia and weakness and difficulty walking. Clinical condition progressively worsened and was characterized by dysphagia, bilateral ptosis, and difficulty breathing. Patient had a two-week history of upper respiratory infection symptoms and was diagnosed with a respiratory viral illness 1 day before symptom onset. Lumbar puncture was done	Yes	GBS

© 2023 Abara WE et al. *JAMA Network Open.*

						but results were not available. Diagnosis: GBS; Treatment: IV immunoglobulin. Patient became hypoxic and required mechanical ventilation. Clinical condition worsened and patient died.		

[a] Age groups instead of exact ages are presented to de-identify decedents

© 2023 Abara WE et al. *JAMA Network Open.*
