# Supplementary material for: Reports of Guillain-Barré Syndrome After COVID-19 Vaccination in the United States
Source: JAMA Netw Open. 2023 Feb 1;6(2):e2253845. doi: 10.1001/jamanetworkopen.2022.53845 (PMC9892957; doi:10.1001/jamanetworkopen.2022.53845)
Supplement: Supplement 2. — Data Sharing Statement [file jamanetwopen-e2253845-s002.pdf]

**Data Sharing Statement**

Abara. Reports of Guillain-Barré Syndrome After COVID-19 Vaccination in the United States. *JAMA Netw Open*. Published online February 01, 2023. doi:10.1001/jamanetworkopen.2022.53845

**Data**
**Data available:** No

© 2023 Abara WE et al. *JAMA Network Open*.
